# Multiple hepatic aneurysms and dry gangrene of fingertips in eosinophilic granulomatosis with polyangiitis: a case report

**DOI:** 10.1186/s13223-020-00484-4

**Published:** 2020-10-15

**Authors:** Eunsil Koh, Noeul Kang, Jin-Young Lee, Duk-Kyung Kim, Young Soo Do, Byung-Jae Lee, Dong-Chull Choi

**Affiliations:** 1 Departments of Medicine, Samsung Medical Center, Sungkyunkwan University School of Medicine, Seoul, Republic of Korea; 2 Departments of Radiology, Samsung Medical Center, Sungkyunkwan University School of Medicine, Seoul, Republic of Korea; 3 Health Promotion Center, Samsung Medical Center, Seoul, Republic of Korea

**Keywords:** Eosinophilic granulomatosis with polyangiitis, EGPA, Aneurysm, Gangrene, Mepolizumab

## Abstract

**Background:**

Eosinophilic granulomatosis with polyangiitis (EGPA) is a systemic necrotizing vasculitis mainly affecting small-sized arteries. Involvement of medium-sized vessels is very rare in EGPA. Here we present the case of a patient with EGPA who showed multiple hepatic aneurysms and distal gangrene.

**Case presentation:**

A known EGPA patient visited to the emergency room (ER) with abrupt squeezing abdominal pain. She had suffered from gangrene in the fingertips of both hands for 1 year because of arterial thrombosis associated with hypereosinophilia. However, her absolute eosinophil count in the ER was 1120 cells/µL. An abdomen-pelvis CT demonstrated subcapsular hematoma in the right hepatic lobe. A celiac angiogram demonstrated multiple sized aneurysms in both hepatic lobes and some aneurysms in S7 and S8 were huge, more than 1 cm in size. The shape of the small aneurysms resembled a string of beads, as in polyarteritis nodosa. Given the clinical situation, emergency embolization was performed. Before this patient visited to the ER, she had been treated with a high dose of systemic corticosteroid, azathioprine, and cyclophosphamide. After addition of mepolizumab, the eosinophil count remained stable state with a near zero percentage of total white blood cell count.

**Conclusions:**

Aneurysm and gangrene resulting from the involvement of medium-sized vessels can occur in EGPA. Destruction of vessels might occur even if eosinophil count is below 1500 cells/µL. If involvement of medium-sized arteries is suspected, thorough investigation to identify the involved organs and prompt management are needed to prevent fatal complications.

## Background

Eosinophilic granulomatosis with polyangiitis (EGPA), formerly named the Churg-Strauss syndrome, is a rare systemic necrotizing vasculitis with eosinophilia. EGPA affects small- to medium-sized blood vessels of various organs [1]. Vasculitis is classified by the size of the predominantly affected vessel, and characteristic manifestations vary depending on the classification of vasculitis [2]. Gangrene or aneurysms, known to be clinical features of medium-sized vasculitis, scarcely present in ANCA-associated vasculitis of small sized vasculitis [3, 4]. Blood eosinophil count of EGPA patients is usually about 5000 cells/µL (reference value < 500 cells/µL) before treatment with corticosteroid or immunosuppressive treatment [5, 6]. The target level of eosinophil count for treatment is usually below 1500 cells/µL. However, we found that rare manifestations could occur even eosinophil count was below 1500 cells/µL, as in the case we present here. We report a rare case of EGPA presenting together with aneurysm and gangrene.

## Case presentation

A 37-year-old woman was presented to the emergency room with abrupt severe right upper abdominal pain. The pain was squeezing and started suddenly after breakfast. The pain was not accompanied by vomiting or fever. The patient had previously been treated at the Department of Medicine in Samsung Medical Center with a diagnosis of EGPA. After successful suppression of blood hypereosinophilia with combination of prednisolone and azathioprine in recent months, she had been in the process of tapering prednisolone down to 15 mg per day. However, after dose reduction, her eosinophil counts slowly increased to 1989 cells/µL and levels of aspartate aminotransferase and alanine aminotransferase were elevated from normal to more than 300 U/L with mild abdominal pain. Consequently, her dose of prednisolone was raised back to 60 mg per day. While waiting for admission for further evaluation, abdominal pain brought her to emergency room.

Five years ago, her first visit was prompted by symptoms of sinusitis, rash and eosinophilia. Three months later, numbness and cutting pain in both upper and lower extremities with fever, myalgia, and asthma developed. A nerve conduction study suggested multifocal sensorimotor neuropathy. Biopsy of the sural nerve revealed ischemic change resulting from vasculitis with prominent eosinophilic infiltration. Although perinuclear anti-neutrophil cytoplasmic antibodies were not detected, the patient was diagnosed as EGPA. After repeated intravenous cyclophosphamide pulse therapy with a high dose of systemic steroids, most symptoms were resolved. Sinusitis, presented as an initial symptom, improved after treatment at an early stage and otolaryngologic complication did not appear afterwards.

**Fig. 1:**
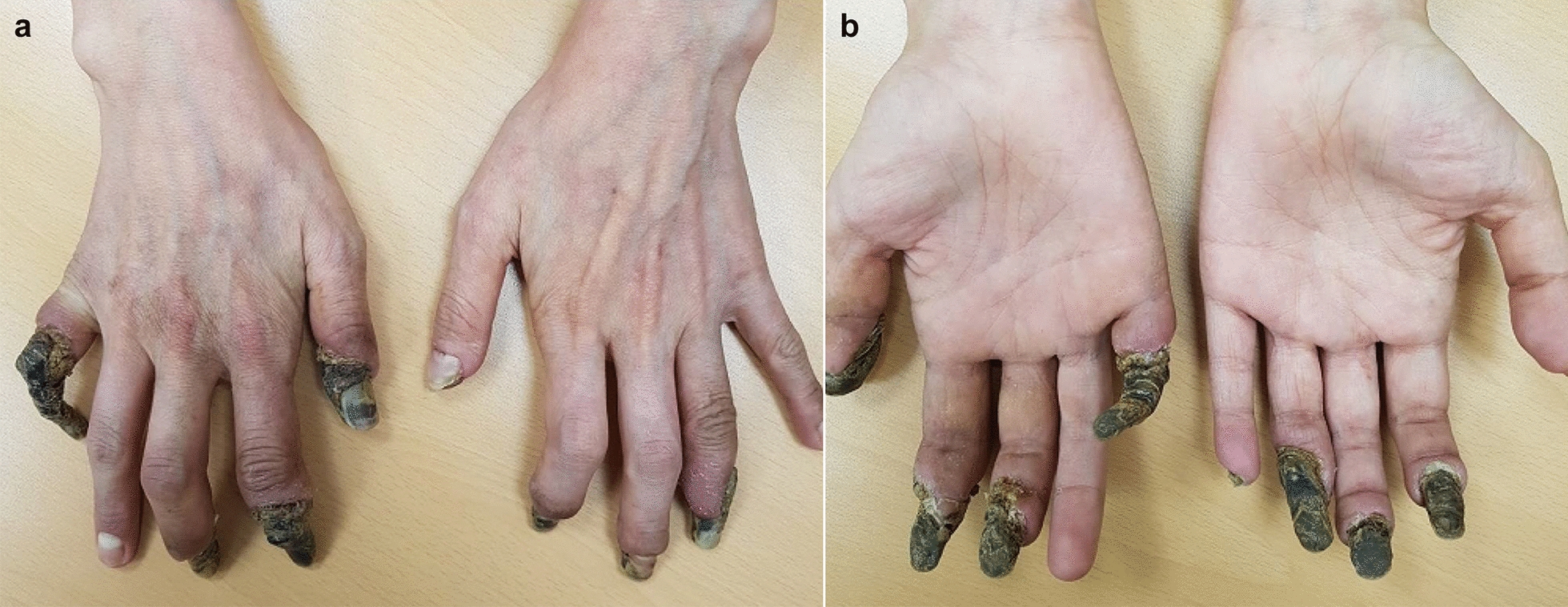
Fingertips of both hands, showing multiple dry gangrene

However, whenever the tapering a dose of steroids was attempted, she developed new symptoms such as myalgia and arthralgia. She even experienced dry gangrene in the fingertips of both hands because of arterial thrombosis when her eosinophil count increased up to 6000 cells/µL (Fig. 1). Duplex scan for arteries and veins indicated the total occlusion of the right distal ulnar artery and left medial forearm ulnar artery. The study also revealed a total occlusion of both mid-anterior tibial arteries, whereas toes of both feet were intact as other vessels of lower extremities were not involved. Hypercoagulability was thought to be caused either directly or indirectly by hypereosinophilia. We recommended mepolizumab, a monoclonal antibody to interleukin-5 used to interrupt eosinophil production, to reduce eosinophil count. But the patient refused it because of cost. We added aspirin and calcium channel blockers after cardiologic consultation. After the affected fingers were dried up, necrotized fingertips were amputated by orthopedic surgeon.

Initial vital signs in the ER showed a blood pressure of 85/45 mmHg, a heart rate of 98 beats per minute with a temperature of 36.1’C, and a respiratory rate of 16 breaths per minute. Physical examination revealed mild tenderness in the right upper abdomen without rebound tenderness. The complete blood count revealed leukocytosis of 17,660 white blood cells/µL. Absolute eosinophil count was 1120 cells/µL. The erythrocyte sedimentation rate was 29 mm/hr, CRP was 3.7 mg/dL and total immunoglobulin E was 107 kU/L.

**Fig. 2:**
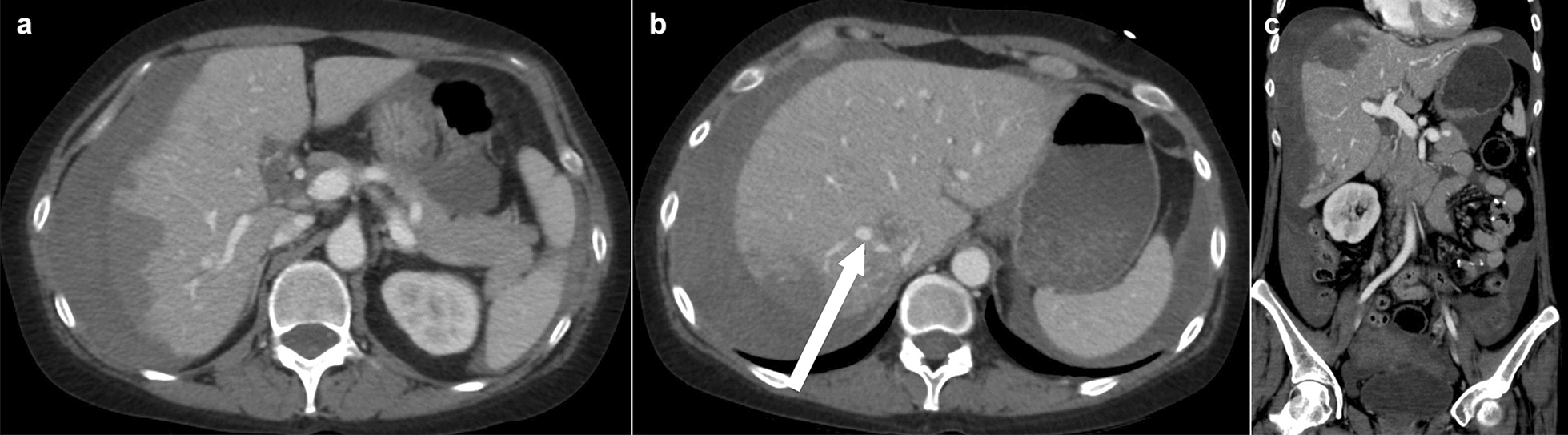
Abdomen-pelvis computed tomography with contrast. **a** Coronal view, demonstrating hepatic subcapsular hematoma in right lobe. **b** Coronal view. Arrow indicates suspicious aneurysmal change. **c** Sagittal reconstructed view, revealing massive amount of hemoperitoneum caused by suspicious aneurysmal rupture

**Fig. 3:**
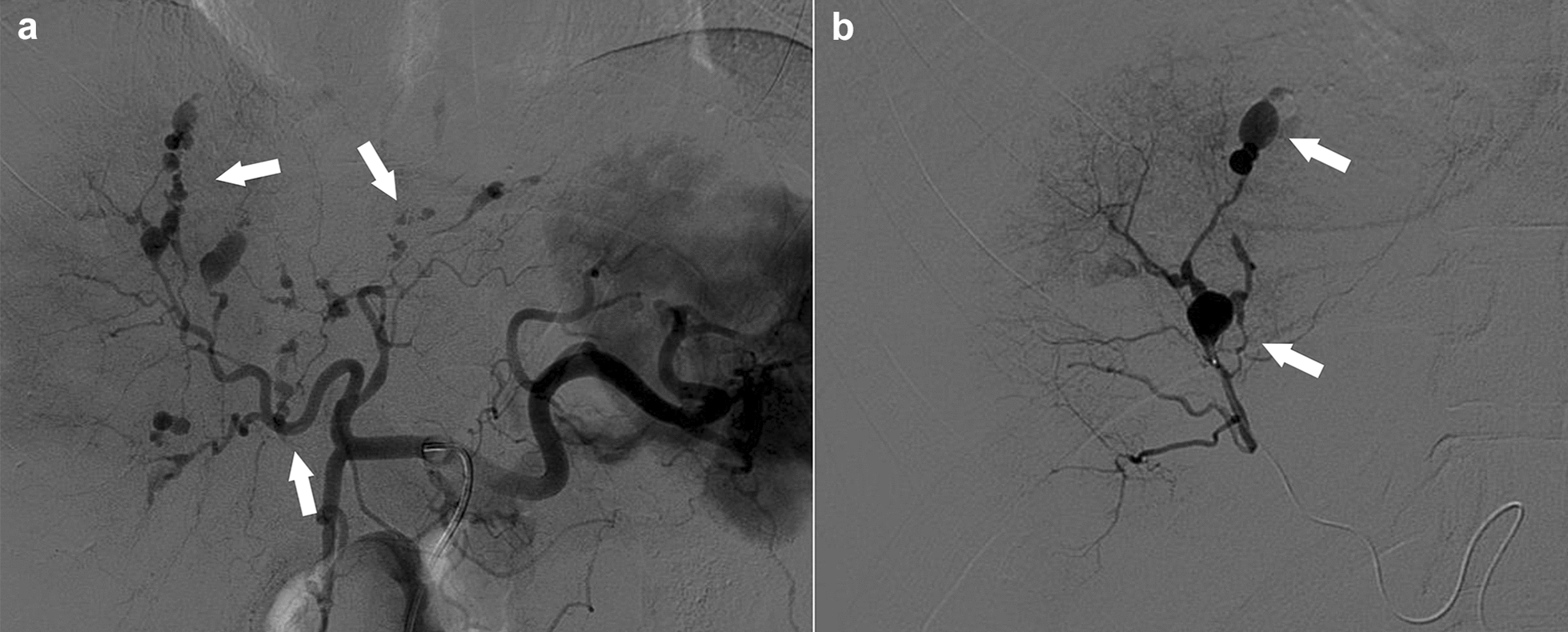
Celiac angiogram before embolization. **a** Angiogram of both lobes, revealing multiple-sized hepatic aneurysms and the appearance of a string of beads. **b** Angiogram of S7 and S8, demonstrating huge aneurysms larger than 1 cm in size

An abdomen-pelvis CT demonstrated subcapsular hematoma in the right hepatic lobe (Fig. 2). Hemoperitoneum probably caused by multifocal hepatic capsular arterial bleeding was also detected. There was no evidence of bowel perforation. In a previous CT scan taken 1 year before, there had been no evidence of any aneurysm changes in the hepatic vessels. A celiac angiogram demonstrated multiple, various sized hepatic aneurysms in both lobes (Fig. 3). The small aneurysms resembled the appearance of a string of beads, characteristically observed in polyarteritis nodosa (PAN). Huge aneurysms larger than 1 cm in size were noticed in S7 and S8. Emergency embolization was performed. The huge aneurysm and the aneurysms with bead-like appearance in S7 and S8 were successfully occluded. Although several small aneurysms in S5, S6 and the left lobe still remained, no further embolization was performed to conserve hepatic function.

Meticulous review of the abdomen-pelvis CT did not reveal evidence of aneurysms in other abdominal organs. After emergency intervention, vital signs became stable and numbness of both hands and feet improved. Based on her clinical course, we strongly recommended to add mepolizumab to control hypereosinophilia and reduce the dose of steroid. After administration of mepolizumab in addition to the previous steroid and cyclophosphamide therapy, eosinophil count became stable and was reduced to a near zero percentage of total white blood cell count.

## Discussion and conclusions

Although EGPA is a systemic necrotizing vasculitis affecting small to medium-sized arteries, significant features of medium-sized vasculitis, such as arterial aneurysm and gangrene, are not common. To the best of our knowledge, this is the first report that both small aneurysms and digital gangrene, known as the characteristics of PAN, developed simultaneously in EGPA. Althogh aneurysm is rare in EGPA, coronary, cerebral, and intrahepatic aneurysm in EGPA have been occasionally reported [4, 7, 8]. A huge hepatic capsular hematoma with unknown origin, reported in other case report [9], looks similar to the image of the abdomen-pelvis CT observed in this case. Hepatic hematoma, cerebral hemorrhage, or other multi-organ hemorrhages in EGPA may be caused by aneurysmal rupture, as demonstrated in this case. An additional biopsy to find direct evidence might be helpful to demonstrate the invasion of EGPA to medium-sized vessels. Nonetheless, further biopsy could not be performed because the patient was unstable due to bleeding, gangrene was dried up and there was no benefit for her already being treated with high dose of steroid and cyclophosphamide.

The pathophysiology of aneurysms and digital gangrene in EGPA is thought to be a combined result of the effects of vasculitis and hypercoagulability induced by hypereosinophilia. Inflammatory cytokines derived from vasculitis and cytotoxic mediators from eosinophil granules may cause endothelial damage directly, leading to the destruction of vessels [10, 11]. The cytotoxicity of eosinophils also induced deconstruction of vessel and provoked hyper-coagulability, resulting thrombus formation in peripheral arteries. This patient did not have any other risk factors for thromboembolic disease or co-morbidities. She had no smoking or drug abuse history. D-dimer was maintained near 2 µg/mL and platelet count was retained between 350,000 and 450,000/µL. The results of a screen for dilute Russell’s viper venom time, antiphospholipid antibody, coagulation factor VIII activity, cardiolipin antibody and anti-beta 2 glycoprotein antibody were negative. Only lupus anticoagulant screening was slightly increased to 66.5 s (normal levels: 32.1–49.8 s). Her mother was a lupus patient, but her anti-nuclear antibody and anti-ds-DNA were negative. There was no evidence of malignancy in chest and abdomen-pelvis CTs as well as in a bone-marrow biopsy.

Another plausible explanation of this case is that PAN overlapped with EGPA. However, it is difficult to confirm this explanation because there are no specific laboratory diagnoses for PAN. Gangrene and aneurysms are the characteristic findings for PAN. However, ruptured hepatic aneurysm and extensive involvement of the entire hepatic arteries in a short period are unusual even in PAN [12, 13]. Asthma and eosinophilia are not manifestation of PAN. Her disease activity was related with hypereosinophilia. This patient has no other indicators of PAN such as livedo reticularis, positive HBV antigen, renal failure and fibrosis or polymorphonuclear neutrophil leukocyte dominant infiltration in the initial biopsy. Liver biopsy could not be performed because it could be dangerous for hepatic vessels in an already vulnerable state. Moreover, she was already being treated with a high dose of steroid and cyclophosphamide which is similar to the treatment of PAN.

This patient was steadily treated with a high dose of steroid and azathioprine. Four cycles of intravenous cyclophosphamide pulse were also given. However, her eosinophil count increased whenever we tried to decrease the dose of steroid. In addition to gangrene, cardiac complications and episcleritis with suspected glaucoma occurred when her eosinophil count increased up to 6000 cells/µL. The ejection fraction of the left ventricle (LV) was decreased to 35%. Global hypokinesia with regional wall motion abnormality, LV dysfunction and minimal pericardial effusion was reported. Furthermore, aneurysmal rupture happened when absolute eosinophil count was just near 1500 cells/µL. Although the ejection fraction, which had previously recovered to normal, slightly decreased to 52% and the creatinine level increased to 3.38 mg/dL which had always been within a normal limit until then, both results were caused by ischemic change because of the massive bleeding of the aneurysmal rupture, and not as complications of EGPA. The finding of echocardiography at this time was the possibility of ischemic heart disease with a lower than normal LV systolic function and regional wall motion abnormality at the inferior wall. The pathologic diagnosis of a kidney biopsy, conducted for the possibility of kidney involvement of EGPA, was interstitial nephritis, and not a glomerular nephritis.

After administration of mepolizumab to reduce the cytotoxicity and hyper-coagulability from hypereosinophilia, her eosinophil count reached a stable state of near zero percentage of total white blood cell count. Mepolizumab is recently used as a steroid-sparing agent for hypereosinophilic disease, such as hypereosinophilic syndrome and EGPA [14, 15]. Although this patient required higher steroid dose due to uncontrolled disease activity, it was difficult for her because she had already taken a high dose of steroid and had experienced CMV colitis before because of the side effects of high dose steroid use. Moreover, mepolizumab in several studies showed not only normalization of eosinophilia but also improvement of Birmingham Vasculitis Activity Score (BVAS) and improvement of the rates of remission of patients with difficult-to-treat EGPA [16, 17]. For this reason, we added mepolizumab with expectations for its steroid-sparing effects and clinical improvement. But more clinical experience is required to assess the effectiveness of biologic agents in EGPA.

In conclusion, the size of the involved vessels may determine commonly affected organs and clinical features in vasculitis. In this case, EGPA can show clinical features of PAN if EGPA invades medium-sized arteries. Small aneurysms and gangrene caused by medium-sized arteries can occur not only in PAN but also in EGPA. Deconstruction of vessels and thrombus formation associated with vasculitis and hypercoagulability may be triggered when eosinophil count is even below 1500 cells/µL in EGPA. If involvement of medium-sized arteries is suspected in EPGA with the signs of distal ischemia or aneurysm, a thorough inspection to identify the involved organs and tighter control of eosinophilia may be vital to prevent fatal complications.

## Data Availability

The data analyzed for the case report are not publicly available due the patient’s request. Data are however available from the corresponding author upon reasonable request and with permission of the legal guardian of the patient.

## Abbreviations

EGPA: Eosinophilic granulomatosis with polyangiitis
ER: Emergency room
CT: Computed tomography
PAN: Polyarteritis nodosa
